# The Au_25_(pMBA)_17_Diglyme Cluster

**DOI:** 10.3390/molecules26092562

**Published:** 2021-04-28

**Authors:** James Armstrong, Chris J. Ackerson

**Affiliations:** Department of Chemistry, Colorado State University, Fort Collins, CO 80523, USA; james.armstrong@colostate.edu

**Keywords:** metal nanoclusters, ligand exchange, surface chemistry

## Abstract

A modification of Au_25_(pMBA)_18_ that incorporates one diglyme ligand as a direct synthetic product is reported. Notably the expected statistical production of clusters containing other ligand stoichiometries is not observed. This Au_25_(pMBA)_17_diglyme product is characterized by electrospray ionization mass spectrometry (ESI-MS) and optical spectroscopy. Thiolate for thiolate ligand exchange proceeds on this cluster, whereas thiolate for diglyme ligand exchange does not.

## 1. Introduction

The ligation shell surrounding protected metal clusters determine properties of the cluster including solubility, capacitance, biocompatibility, reactivity toward ligand exchange, and catalytic capability [1,2,3,4,5]. Ligand shells of thiolate protected gold clusters can be modified by ligand exchange reactions. When a mixed ligand shell is produced, the product is inexact, representing a statistical distribution of both ligands, summing to the total number of ligands [6,7]. Mixed ligand shells with such a statistical distribution of two or more ligand types can also be produced when multiple ligand types are used in cluster synthesis [8].

A few approaches can produce monofunctional gold clusters. These are desirable for bio-labelling, to create bioconjugates of exclusively 1:1 cluster:biomolecule stoichiometry [9,10]. Production of monofunctional clusters and/or nanoparticles can proceed by either of two approaches. One is to purify monofunctional clusters from a statistical distribution which contains multi-functional clusters [10]. Alternatively, a solid phase approach can produce clusters or nanoparticles that are monofunctional [11]. Both approaches represent a substantial and labor-intensive endeavor compared to a typical one-pot direct synthesis of gold nanoclusters.

Previously, our group reported that the 2-phenylethanethiol (PET) protected nanocluster Au_20_(PET)_15_ could be assembled into dimers linked by diglyme. The clusters must be synthesized in diglyme as a solvent to observe this behavior [12]. The Au_20_(PET)_15_(diglyme)Au_20_(PET)_15_ dimers are in a dynamic equilibrium with Au_20_(PET)_15_ protomers. The finding of glyme linked clusters is interpreted as glyme making direct and dative (weak) bonds to gold. The overall finding was surprising due to the high strength of the Au-S bond compared to the bonding strength of diglyme to gold [13]. The overall interpretation is that when diglyme is present as solvent (in high molar quantities), that it can compete with thiolates for bonding to the surface of gold clusters.

## 2. Results and Discussion

Au_25_(SR)_18_ is the benchmark thiolate protected gold nanocluster [14]. It is preferentially produced in gold cluster synthesis due to its high stability against thermal etching [15]. This has made it a widely used molecule to establish aspects of gold nanocluster synthesis, ligand exchange, and applications; many properties that are found initially for Au_25_(SR)_18_ are later shown to be common to thiolate protected gold clusters in general [2,16,17].

In this work, we show a modification of p-mercaptobenzoic acid (pMBA) protected Au_25_(pMBA)_18_ with a diglyme ligand, resulting in a cluster formulated as Au_25_pMBA_17_diglyme. Spectroscopic and mass spectrometric analysis establish the existence of this cluster. Ligand exchange with incoming a thiol ligand was attempted, under the hypothesis that the diglyme ligand would preferentially exchange. Unexpectedly, diglyme does not exchange with a thiol ligand in any exchange condition, whereas other thiolate ligands do exchange.

To synthesize the cluster, a 125 mL Erlenmeyer flask was charged with 24 mL of 100 mM pMBA in 0.3 M NaOH in water and a stir bar. Addition of 8 mL 100 mM HAuCl_4_ in diglyme was added dropwise. This creates a clear, yellow solution which is stirred for 30 min before 0.500 mL of 10 mM sodium borohydride in diglyme is slowly drip added to the solution over 1 min, resulting in a color change to a deep red color. This solution is collected and diluted from 8 mL aliquots to 50 mL with diglyme, 1 mL of ammonium acetate is added. The product is collected by centrifugation. This results in an aqueous biphasic system, with the reaction products concentrated in the bottom phase at approximately 2 mL volume and black in color. The black, aqueous phase is then separated using polyacrylamide gel electrophoresis (PAGE.) This typically reveals three products (bands) (Supplemental Figure S1), with the bottom band appearing as a deep red color. All products are thought to be nanocluster sized, however this manuscript focuses on the bottom (red) product because it could be characterized by ESI-MS, whereas the other products could not be characterized by ESI-MS, presumably due to harsh ionization conditions.

The red product is collected by excising the band from of the PAGE gel. The excised gel containing the product is powdered with a mortar and pestle and eluted from the gel by an overnight soak in 10 mL of water. Gravity filtration through a 150 mm filter separates the soluble product from the insoluble gel. Products appeared to be stable out to at least two weeks under ambient storage conditions, given the timeline of work of these experiments.

The purified product was analyzed by electrospray ionization—mass spectrometry (ESI-MS). Triethylammonium counterions were added because they improve spectra in ESI-MS of gold nanoclusters [18].

**Figure 1:**
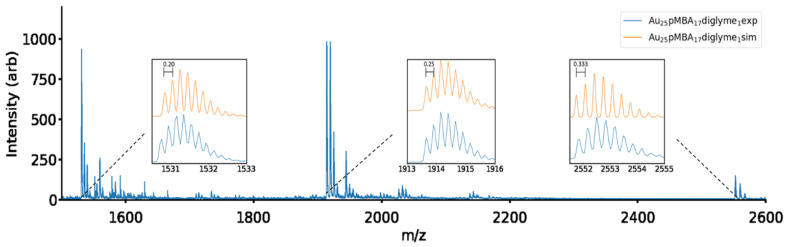
ESI-MS spectra Au_25_pMBA_17_diglyme, with nine protonated carboxylic acid groups, and eight deprotonated, shows three peak groupings. The peak spacing indicates charge states of −5, −4, and −3, respectively. All three parent peaks can be explained by a Au_25_pMBA_17_ diglyme cluster. Subsequent peaks relate to sodium adducts of the cluster through the carboxylic ligand group.

ESI-MS of the purified cluster is shown in Figure 1. There are three apparent groups of peaks. The distance between peaks each grouping allows inference of the species charge, allowing a total mass calculation. The three peak groupings correspond to three charge states (−3, −4, and −5) of a nanocluster of atomic mass 7655 a.u. This mass corresponds well with a cluster formula of Au_25_pmba_17_ diglyme. That cluster has a mass of 7655 a.u. when eight of the ligands are deprotonated. Figure 1, inset, shows the simulated ESI mass spectrum for Au_25_pmba_17_ diglyme without eight protons, comparing it to the experimental spectrum. The possibility of other combinations of Au and pMBA were also considered. Notably, we cannot assign this spectrum to the known Aux(SR)y clusters of nearby formulae, including Au_25_pMBA_18_ (mass 7682 a.u.), Au_23_pMBA_16_ (6981 a.u.), or Au_24_pMBA_20_ (7791 a.u.) [18]. Full spectra are available in Supplemental Figure S2, with peak assignments in Supplemental Table S1. Supplemental Table S2 tabulates the expected masses for each of these clusters and their possible protonation states. Some calculated nanocluster assignments are explored further in Supplemental Table S2. Based on the mass spectrum of Figure 1, the product is assigned as Au_25_(pMBA)_17_ diglyme.

**Figure 2:**
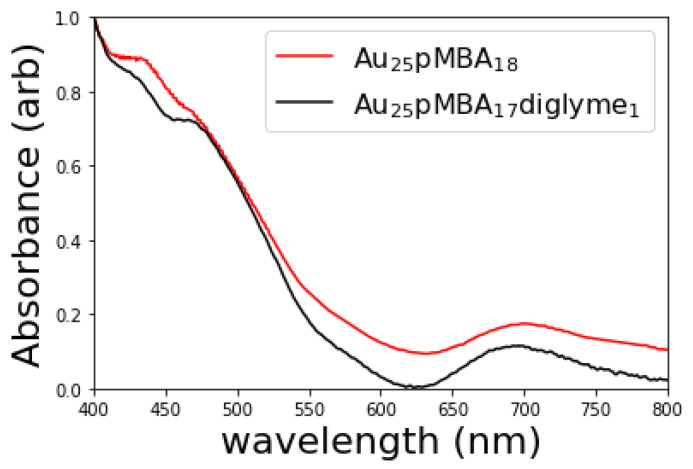
UV–VIS for Au25pMBA_17_diglyme1 (black) is comparable to the UV–VIS spectrum for Au25pMBA_18_ (red), reproduced from [19].

Supporting this assignment is that the linear absorbance spectrum of Au_25_(pMBA)_17_diglyme is similar, but not identical to the Au_25_(pMBA)_18_ spectrum (Figure 2). The Au_25_(pMBA)_17_ diglyme spectrum exhibits features at around 410, 440, and 680 nm. These features are similar to the Au_25_(SR)_18_ nanocluster, which shows similar features around 430, 470, and 680 nm [19]. These linear absorption spectral features are attributed to the geometry of Au in a cluster [20]. This linear absorbance spectrum, therefore, suggests that the cluster consists of 25 gold atoms with 18 ligands in a similar geometric configuration to the very well-studied Au_25_(SR)_18_.

We considered that the differences in the linear optical spectrum could be explained as different oxidation states of Au_25_(SR)_18_ clusters. Au_25_(SR)_18_ clusters are known to be stable and isolable in +1/0/−1 oxidation states. This oxidation state is relative to the core gold atoms and is independent of ligand charge. Generally, the oxidation state of a cluster can be determined most reliably by electrochemical means, such as a differential pulse voltammogram to establish the potentials at which the cluster (with a given ligand shell) is in each of the oxidation states. Then a resting potential measurement of a sample can establish the oxidation state of the clusters in the sample. For water-soluble gold clusters, however, electrochemical measurements cannot be made reliably.

Optical spectra of Au_25_(SR)_18_ nanoclusters are reported in +1, neutral, and −1 oxidation states, and show distinguishing features. The linear absorption spectrum for Au_24_(pMBA)_17_diglyme appears most similar to known spectra of +1 oxidation state of Au_25_(SR)_18_ at the features around 430 and 470. However, the Au_24_(pMBA)_17_diglyme spectrum around 680 nm more closely resembles the −1 oxidation state of Au_25_(SR)_18_ [15,21]. Therefore, we cannot conclusively attribute the oxidation state based on the spectrum; The differences in the spectrum between the Au_25_(pMBA)_18_ (which can be assigned as oxidation state 0 based on the spectrum) and Au_25_(pMBA)_17_diglyme are likely due to the presence of diglyme in the ligand shell.

The finding of a synthesis that produces a monofunctional Au_25_pMBA_17_diglyme cluster prompted study of ligand exchange characteristics. Incoming ligand feeds of 3-mercaptopropionic (3-MPA) acid to pMBA were tested from a 1:1 ratio of incoming ligand:cluster, up to 10,000:1 (Supplemental Figures S3 and S4). Solution pH was varied from 6 to 11; At pH values below 6 the clusters are insoluble. Temperatures from room temperature to 60 °C were attempted. In all cases, ESI-MS spectra of the ligand exchange products revealed exchange of pMBA for 3-MPA, but no exchange of diglyme for 3-MPA. Interestingly, synthesis was not viable under similar conditions with other glymes, and exchange of diglyme for new incoming glyme’s was not observed.

**Figure 3:**
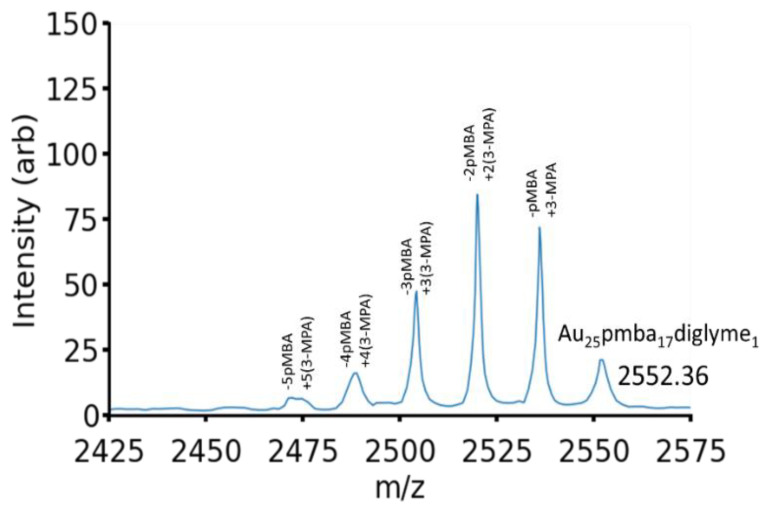
ESI-MS spectra of ligand exchange of Au25pMBA_17_diglyme_1_ with 3-mercaptopropionic acid. The furthest right peak can be attributed to the −5 charge state of Au25pMBA_17_diglyme_1_. Each subsequent peak to the left correlates to the exchange of a pMBA ligand for an incoming 3-MPA ligand.

Figure 3 shows the ESI-MS spectrum of a ligand exchange reaction executed with a 1000-fold excess of 3-MPA for 30 min at room temperature. This represents a typical result. In Figure 3, each peak can be attributed to the exchange of a pMBA ligand for a 3-MPA ligand, as annotated in the figure. In this reaction condition, we observe the exchange of up to five ligands, with two ligand-exchange appearing as the dominant product. There is no evidence of diglyme exchange.

Since the binding energy of thiolate ligands on gold is much more favorable than the binding energy of diglyme on gold, the absence of diglyme for thiol exchange is surprising [12]. We can propose some mechanistic reasons for the absence diglyme exchange. For instance, most ligand exchange on gold clusters proceeds by an associative mechanism which requires solvent exposed gold atoms [2,22]. It may be the case that diglyme ligands are bonded to gold atoms that have no solvent exposure, mechanistically blocking ligand exchange. Diglyme may also be bound in a multidentate manner, whereas the thiolate ligands are monodentate; Multidentate binding of diglyme may interfere mechanistically with ligand exchange.

One ongoing interest is deciphering ligand regiochemistry on gold nanoclusters [17]. Regiochemical control of ligand location on gold nanoclusters is challenging because of the facile nature of inter-particle ligand exchange [7]. The inability of diglyme to exchange with thiolate ligands may represent a step toward improved regiochemical control of ligand locations on gold nanoparticles.

Interestingly, this system does not show any evidence for assembly into dimers or larger structures through the diglyme ligand. This was unexpected given our previous observations of Au_20_(PET)_15_ dimers linked through a diglyme molecule. The present finding implies an enhanced role of pi-pi stacking between Au_20_(PET)_15_ nanoclusters playing a role in assembly, as was initially suggested by IR spectroscopy in our initial report [12]. The carboxylic acid group functional groups on pMBA could prevent this interaction occurring here through steric hindrance or the repulsion of similar surface charges between nanoclusters.

## 3. Materials and Methods

### 3.1. Materials

Gold(III) chloride trihydrate (HAuCl_4_·3H_2_O, ACSreagent, >49.0% Au basis), sodium borohydride (NaBH_4_, powder, >98.0%), ammonium acetate (NH_4_OAc, ACSreagent, >97.0%), para-mercaptobenzoic acid (pMBA, >95.0%), 3-mercaoptopropionic acid (≥99%), (sodium hydroxide (NaOH, pellets, certified ACS), 2-Amino-2-(hydroxymethyl)-1,3-propanediol (Tris base, ≥99.9%), boric acid (H_3_BO_3_, ≥99.5%), glycerol (C_3_H_8_O_3_, ≥99.5%), calcium chloride dehydrate (CaCl_2_·2H_2_O, crystalline), ethylenediaminetetraacetic acid (EDTA, powder, >99.4%), and diethylene glycol dimethyl ether (diglyme, anhydrous, 99.5%) were obtained from Sigma-Aldrich, St. Louis, MO, USA.

### 3.2. Synthesis of Au_25_(pMBA)_17_diglyme

First, 8 mL of 100 mM HAuCl_4_*3H_2_O in diglyme was added to 24 mL of 100 mM p-MBA in 0.3 M NaOH in a 250 mL Erlenmeyer flask. The reaction mixture was stirred for 30 min at 0 °C in an ice bath. The reaction mixture underwent a color change from transparent red to transparent yellow indicating the reduction of gold in the formation of Au(I)-[SR-Au(I)]x chains. Addition of 10 mM NaBH_4_ in diglyme, by 100 µL aliquots, 1 per minute for 5 min, caused a color change to dark brown/black indicating the formation of nanoclusters. Dilution of 8 mL aliquots of reaction mixture to 50 mL was done with diglyme, followed by 1 mL addition of 5 M ammonium acetate. Centrifugation caused separation into solid black, viscous black layer, and a colorless layer. The viscous layer is separated by TBE-PAGE, giving three products A, B, and C. Polyacrylamide gel electrophoresis (PAGE) is run using a buffer of 89 mM Tris base, 89 mM boric acid, and 2 mM EDTA (TBE). PAGE is run using a VWR power source at a constant voltage of 125 V for 3:00 h. Samples are mixed 1:1 by volume with 50/50% b/v glycerol:water to assist loading into gel.

### 3.3. Ligand Exchange of Au25pMBA17diglyme

An Erlenmeyer flask is charged with 0.1 mM Au25pMBA_17_diglyme. Appropriate volume of 3-mercaptopropionic acid for the intended ratio of incoming ligand:cluster(1:1–10,000:1) is added and allowed to stir at the appropriate temperature for 30 min. The reaction is quenched by dilution of 8 mL aliquots of reaction mixture to 50 mL was done with diglyme, followed by 1 mL addition of 5 M ammonium acetate. Samples are collected by centrifugation and prepared for ESI-MS analysis.

### 3.4. Electrospray Ionization-Mass Spectrometry

Samples were prepared by dissolving clusters in 1 mL of 10 mM triethylammonium buffer. These samples were precipitated in 50 mL ethanol with 1 mL of 5 M ammonium acetate. Samples were dissolved and precipitated three times in this manner to wash excess diglyme and salts. mass spectrometry-electrospray ionization (ESI-MS) was run using an Agilent Technologies G6220A instrument run in negative ionization mode. Source parameters include: Gas temp 150 °C, vaporizer 120, gas flow(L/min) 6.0, nebulizer (psi) 18, and V_Charge_ 2000. The scan rate was 1.34. Samples were run at a concentration of 0.01 mg/mL in water.

## 4. Conclusions

In conclusion, the highlights of this work include the synthesis of a cluster with a single diglyme ligand that does not participate in subsequent self-assembly. Since these clusters are water-soluble and singly functionalized, they may represent the beginnings of a more straightforward pathway for synthesizing the mono-functional clusters commonly used in bio-labeling [23].

## Data Availability

Raw data is available from authors upon request.

## Supplementary Materials

Supplemental Figure S1: TBE-PAGE separation of nanocluster products; Supplemental Figure S2: Full ESI-MS spectra; Supplemental Figures S3 and S4: Ligand exchange with 3-mercaptopropionic acid;-S4, Supplemental Table S1: Peak Assignments from Full Spectra; and Supplemental Table S2: Observed nanocluster mass and potential assignments.
