# Supplementary material for: The Au25(pMBA)17Diglyme Cluster
Source: Molecules. 2021 Apr 28;26(9):2562. doi: 10.3390/molecules26092562 (PMC8124888; doi:10.3390/molecules26092562)
Supplement: Supplementary file 1 [file molecules-26-02562-s001.zip › molecules-1197872-supplementary.pdf]

Supplemental Information

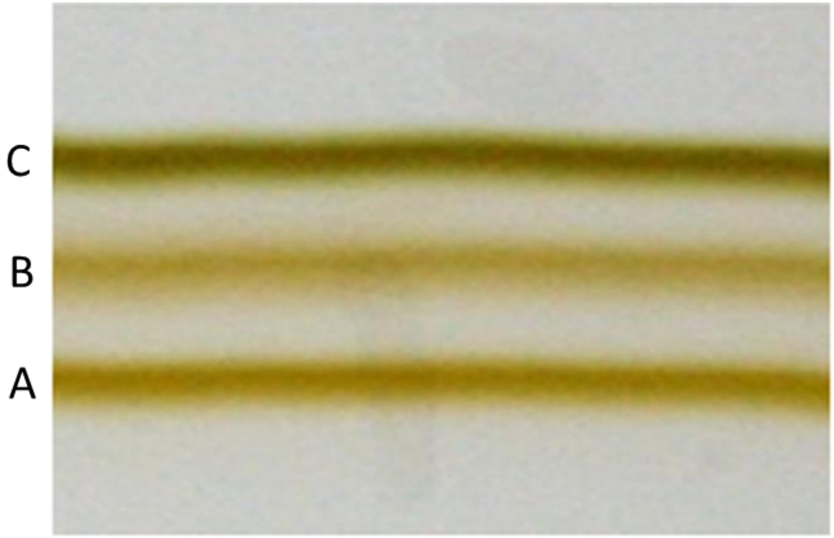

Supplemental Figure S1: TBE-PAGE separation of synthetic products A, B, and C. A has been identified as $Au_{25}(pMBA)_{17}diglyme_1$ and is the focus of this work, B and C are expected to be a larger cluster but has yet to be identified.

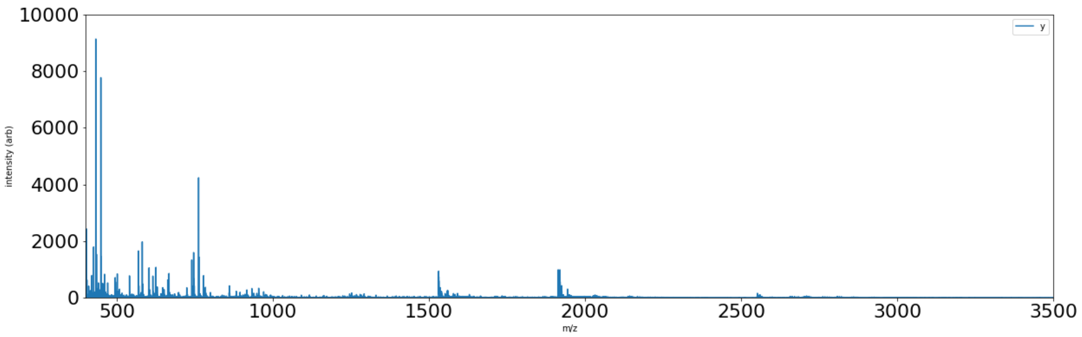

Supplemental Figure S2: Full spectra for $Au_{25}(pMBA)_{17}diglyme_1$ analysis.

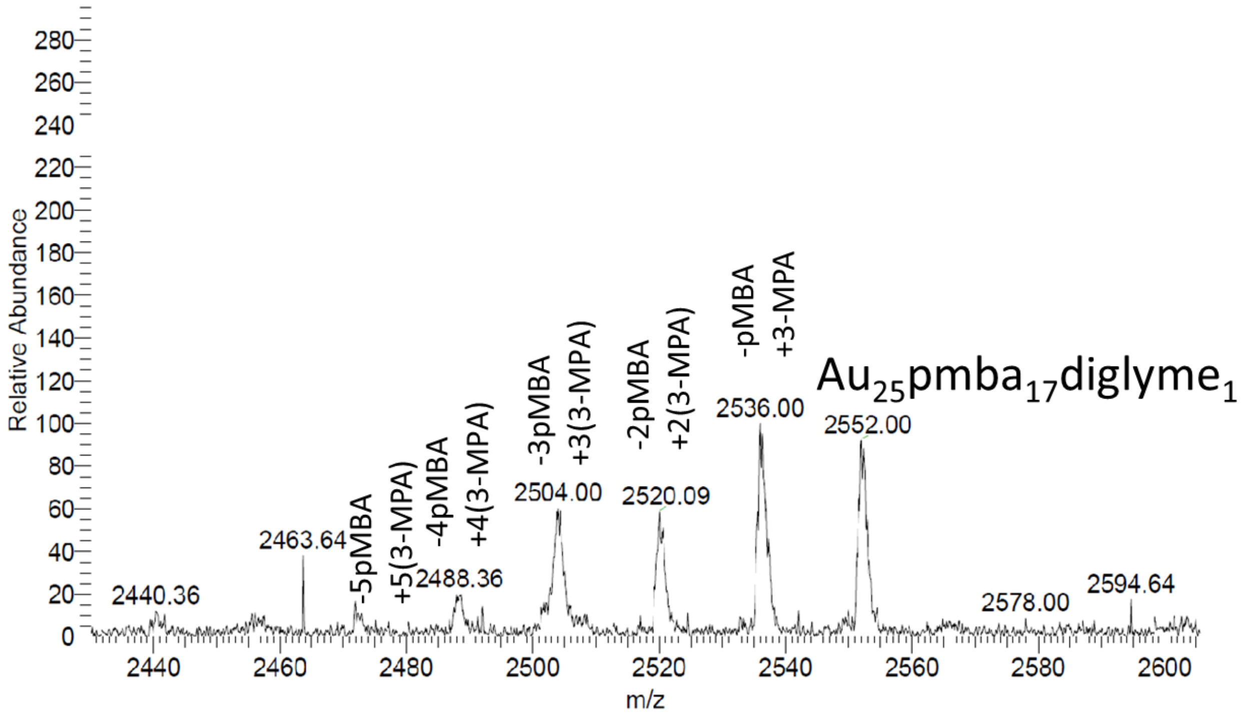

Supplemental Figure S3: Ligand exchange with 3-mercaptopropionic acid as the incoming ligand. This was done at a 5000:1 ligand:cluster ratio, and pH of 6.

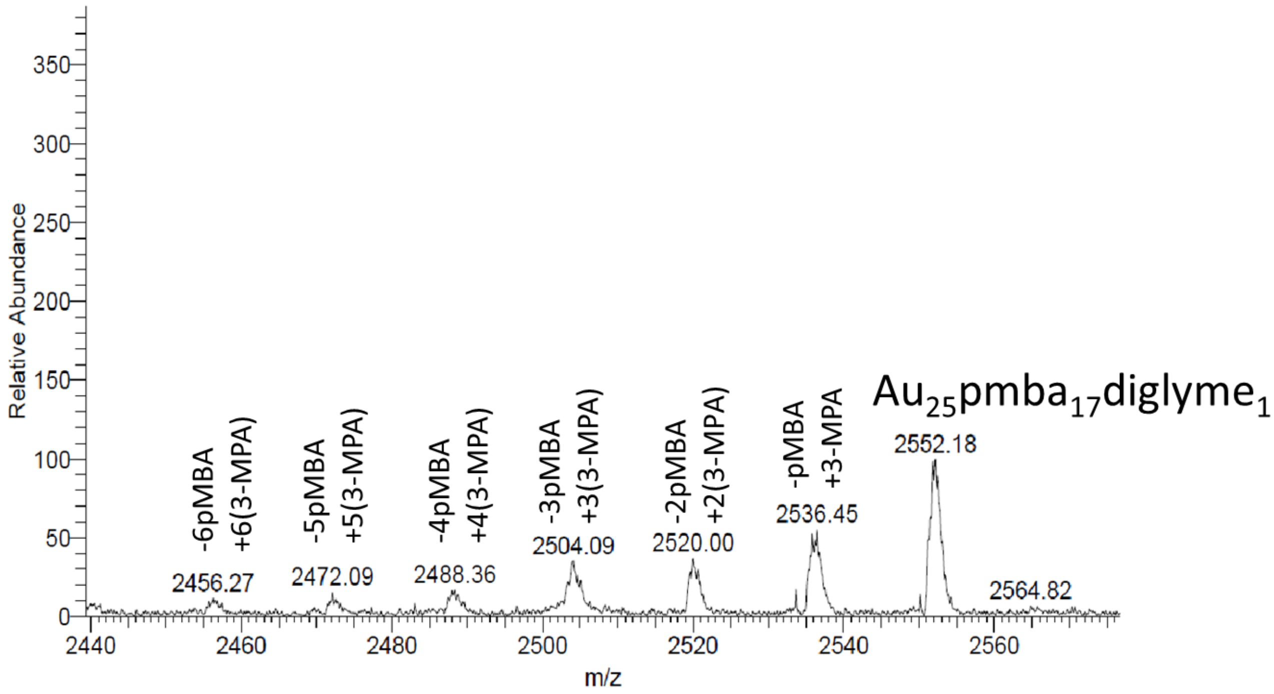

Supplemental Figure S4: Ligand exchange with 3-mercaptopropionic acid as the incoming ligand. This was done at a 1000:1 ligand:cluster ratio, and pH of 6.

Supplemental Table S1: Peak assignments from Full Spectra

Parent Peak(m/z)	Charge state	Chemical Formula
2551.8627	-3	$Au_{25}pmba_{17}diglyme_1$
2559.1563	-3	$Au_{25}pmba_{17}diglyme_1+Na^+$
2566.5027	-3	$Au_{25}pmba_{17}diglyme_1+2Na^+$
1913.6447	-4	$Au_{25}pmba_{17}diglyme_1$
1919.1587	-4	$Au_{25}pmba_{17}diglyme_1+Na^+$
1924.8815	-4	$Au_{25}pmba_{17}diglyme_1+2Na^+$
1530.7584	-5	$Au_{25}pmba_{17}diglyme_1$
1535.3315	-5	$Au_{25}pmba_{17}diglyme_1+Na^+$
1539.7316	-5	$Au_{25}pmba_{17}diglyme_1+2Na^+$

Supplemental Table S2: Table of the observed nanocluster mass and potential assignments.

Atom Counts								
Au (196.97a.u.)	pMBA -H (153.182a.u.)	pMBA -2H (152.182a.u.)	diglyme (134.17a.u.)	mass	mass/2	mass/3	mass/4	mass/5
25	18	0	0	7681.526	3840.763	2560.509	1920.382	1536.305
25	0	18	0	7663.382	3831.691	2554.461	1915.846	1532.676
25	17	0	1	7662.514	3831.257	2554.171	1915.629	1532.503
25	0	17	1	7645.378	3822.689	2548.459	1911.345	1529.076
25	9	8	1	7654.45	3827.225	2551.483	1913.613	1530.89
24	0	20	0	7770.76	3885.38	2590.253	1942.69	1554.152
23	16	0	0	6981.222	3490.611	2327.074	1745.306	1396.244
Observed Nanocluster				7655.5		2551.843	1913.666	1530.749
